# Severe thrombocytopenia in a patient with inosine triphosphatase (ITPA)–*CC* genotype caused by pegylated interferon (IFN)-α-2a with ribavirin therapy: a case report

**DOI:** 10.1186/1756-0500-7-141

**Published:** 2014-03-12

**Authors:** Weimin Jiang, Hisashi Hidaka, Takahide Nakazawa, Hiroyuki Kitagawa, Wasaburo Koizumi

**Affiliations:** 1 Department of Infectious Disease, Huashan Hospital, Fudan University, Shanghai, China; 2 Department of Gastroenterology, Kitasato University School of Medicine, East Hospital, 2-1-1 Asamizodai, Minami-ku, Sagamihara, Kanagawa 252-0380, Japan

**Keywords:** Hepatitis C, *ITPA*, Pegylated interferon, Thrombocytopenia

## Abstract

**Background:**

Pegylated interferon combined with ribavirin treatment is an effective therapy for chronic hepatitis C viral infection. However, pegylated interferon combined with ribavirin is associated with various adverse reactions. Severe thrombocytopenia is a life-threatening side effect of interferon therapy that can lead to bleeding. It is generally understood that the inosine triphosphatase-CC genotype does not have a significantly lower reduction by pegylated interferon combined with ribavirin in the mean platelet counts compared with the *AA*/*CA* genotype. We report a case of severe thrombocytopenia that developed in a patient with chronic hepatitis C treated with pegylated interferon combined with ribavirin in spite of having the inosine triphosphatase-CC genotype.

**Case presentation:**

A 57-year-old female had been diagnosed as having HCV infection in 2008. The inosine triphosphatase gene showed one single nucleotide polymorphism *(rs1127354) C/C* (major homozygous) and the *IL28B* gene showed single nucleotide polymorphism *(rs8099917 T/T, rs11881222 T/T)* (major homozygous). The patient was treated with pegylated interferon 180 μg once a week combined with ribavirin 600 mg per day from April 2011. The hepatitis c virus ribonucleic acid turned negative 9 weeks after treatment with pegylated interferon combined with ribavirin. During the therapy, the platelet count remained above 8.0 × 10^4^/μl for about 9 months. In January 2012, the platelet count was 6.8 × 10^4^/μl. In February 2012, the 44th week from the beginning of the treatment, a sudden decrease in the platelet count to 0.8 × 10^4^/μl was observed. After prednisolone was administered, the platelet count increased. Finally the platelet count had risen above normal range.

**Conclusion:**

We should pay careful attention in the differential diagnosis for patients with the inosine triphosphatase-CC genotype because, although rare, severe thrombocytopenia could occur.

## Background

Chronic infection with hepatitis C virus (HCV) affects up to 170 million individuals worldwide [1] and may lead to progressive hepatic fibrosis and cirrhosis, with risk of liver failure and hepatocellular carcinoma. Pegylated interferon (PEG-IFN) combined with ribavirin (RBV) treatment (PEG-IFN/RBV) is an effective therapy for chronic HCV infection. However, PEG-IFN/RBV is associated with various adverse reactions. Some of these side effects are mild, such as flu-like symptoms. Some others can be very severe and even lead to lethal consequences [2]. Severe thrombocytopenia is a life-threatening side effect of interferon therapy that can lead to bleeding, and even death, if diagnosis and treatment are not adequately given. Therefore, how to identify the possibility of thrombocytopenia early is critical for patients treated with PEG-IFN/RBV.

Recently, genome-wide association studies have identified that the genetic variant of *rs1127354* single nucleotide polymorphisms (SNPs) in the inosine triphosphatase (*ITPA*) gene, which encodes a protein that hydrolyses inosine triphosphate (ITP), has been found to be associated with thrombocytopenia. The *ITPA-AA/CA* genotype was independently associated with a higher degree of reduction in platelet counts at 4 weeks as well as protection against the reduction of hemoglobin in patients treated with PEG-IFN/RBV [3-5], while the *CC* genotype had significantly lower reduction in the mean platelet counts compared with the *AA/CA* genotype [5]. Here, we report a case of severe thrombocytopenia that developed in a patient with chronic hepatitis C treated with PEG-IFN-α2a plus ribavirin in spite of having the *ITPA-CC* genotype.

## Case presentation

A 57-year-old female had been diagnosed as having HCV infection in 2008 in another hospital. Laboratory test showed that her serum and anti-HCV, and HCV ribonucleic acid (RNA) were positive in October 2010 at Kitasato University East Hospital. The patient’s laboratory findings prior to receiving treatment, in March 2011, are shown in the Table 1. The *ITPA* gene showed one *single nucleotide polymorphism (SNP) (rs1127354) C/C* (major homozygous) and the *IL28B* gene showed *SNP (rs8099917 T/T, rs11881222 T/T)* (major homozygous) [5].

**Table 1:** The patient’s laboratory findings prior to receiving treatment

WBC	5800	/μL	CRP	<0.10	mg/dl
RBC	404 × 10^4^	/μL	LDH	238	U/L
Hb	13.5	g/dL	UA	5.8	mg/dL
Plt	16.7 × 10^4^	/μL	BUN	12.6	mg/dL
PT	11.7	sec	Cr	0.52	mg/dL
AST	41	IU/L	Na	141	mEq/l
ALT	46	IU/L	K	3.8	mEq/l -
TP	7.6	g/dL	HBsAg	(−)	-
Alb	4.4	g/dL	HCVAb	(+)	-
TBIL	0.5	mg/dL	HCV-RNA	6.3	LogIU/ml
ALP	248	IU/L	Genotype	1B	
γ-GTP	20	IU/L			

WBC, white blood cells; RBC, red blood cells; Plt, platelets; PT, prothrombin time; AST, aspartate aminotransferase; ALT, alanine aminotransferase; TP, total protein; Alb, albumin; TBIL, total bilirubin; ALP, alkaline phosphatase; γ-GTP, gamma glutamyl transpeptidase; CRP, C-reactive protein; LDH, lactate dehydrogenase; UA, uric acid; BUN, blood urea nitrogen; Cr, creatinine; Na, natrium; K, potasium; HBsAg, hepatitis B surface antigen; HCV, hepatitis C virus antibody; HCV-RNA, hepatitis C virus ribonucleic acid.

**Figure 1:**
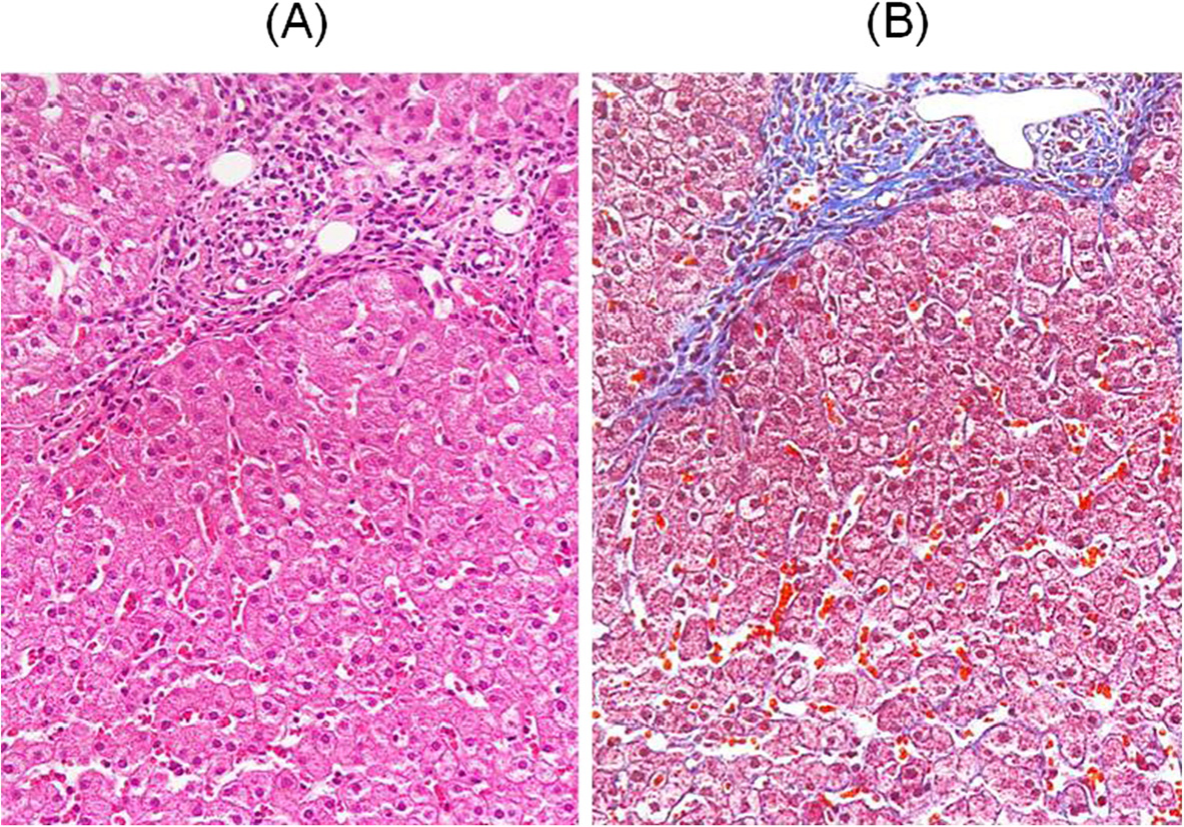
Liver biopsy (A: Hematoxylin and Eosin stain × 100; B: Masson trichrome stain × 100): fibrosis grade 3 and inflammatory activity grade 1 in the Metavir classification, Histologic activity index score: 1-1-3-3.

She had a history of a little alcohol consumption and of smoking 20 cigarettes per day for longer than 30 years. Her height was 156.3 cm, and her body weight was 57.5 kg. Her mother had a history of hypertension. The patient had no history of ever having a blood transfusion. The ultrasonographic examination showed that the patient’s liver, spleen, and pancreas were normal, but she had gallstones. The liver biopsy revealed chronic active hepatitis (fibrosis grade 3 and inflammatory activity grade 1) in the Metavir classification and a hepatic activity index score of 1-1-3-3 (Figure 1).

**Figure 2:**
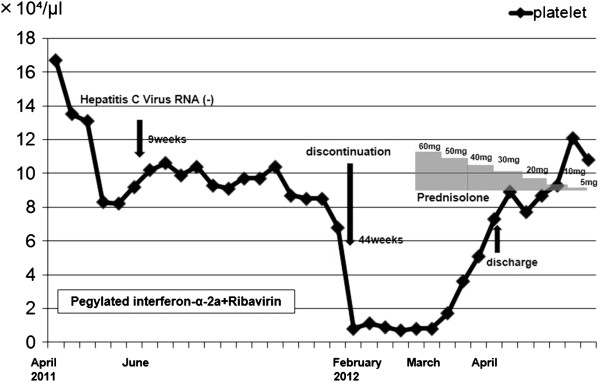
Clinical course of the patient.

The patient was treated with PEG-IFN-α-2a 180 μg once a week combined with ribavirin 600 mg per day from April 2011. The HCV RNA turned negative 9 weeks after treatment with PEG-IFN-α-2a. During the therapy, the platelet count remained above 8.0 × 10^4^/μl for about 9 months. In January 2012, the platelet count was 6.8 × 10^4^/μl. In February 2012, the 44th week from the beginning of the treatment, a sudden decrease in the platelet count to 0.8 × 10^4^/μl was observed (Figure 2). Severe thrombocytopenia caused by the PEG-IFN-α-2a therapy was suspected. Therefore, the PEG-IFN-α-2a and ribavirin were discontinued. The patient was then admitted to our hospital.

On admission, the blood pressure, temperature and pulse of the patient were normal. Her white blood cell and neutrophil values were also in the normal range. And there was no evidence of bacterial, viral, or other microorganism infection. Purpura were noted on both lower limbs and her trunk. The white blood cell count was 3000/μl, the red blood cell count was 3.14 × 10^6^/μl, hemoglobin was 11.2 g/dl, but the platelet count had decreased to 1.1 × 10^4^/μl. The liver function and coagulation function were normal. But the PAIgG was elevated to 180 ng/L (normal range, 9-25 ng/L).

**Figure 3:**
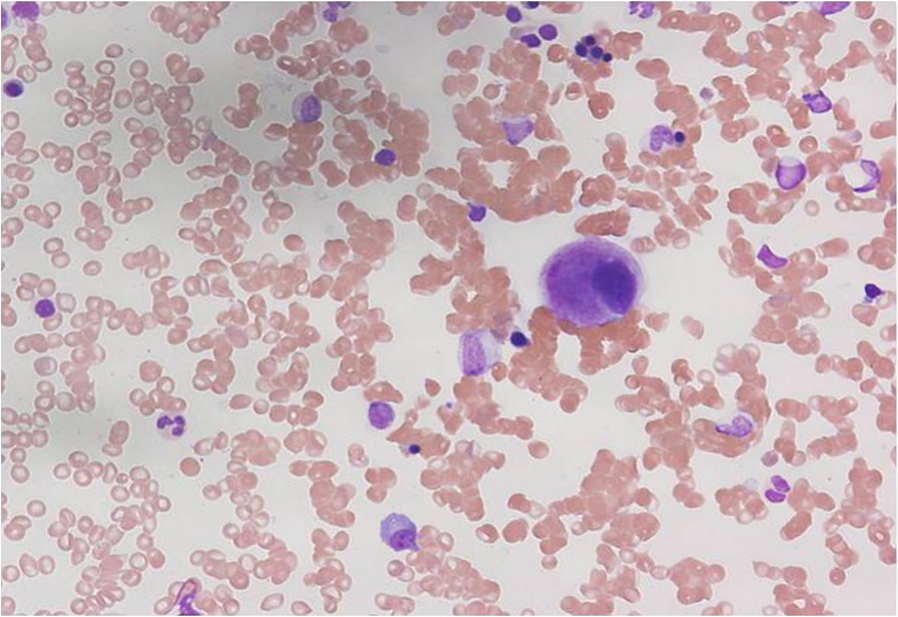
Bone marrow specimen (May-Geimsa stain × 400): The specimen showed myelocyte hypoplasia, especially in megakaryocyte, and no atypical cells.

A bone marrow aspiration examination was performed to investigate the etiology of the thrombocytopenia. The specimen revealed that there was hypoplasia of myelocytes (especially in megakaryocytes) but no myeloblasts, which did not suggest any evidence of bone marrow disease (Figure 3).

Based on these results, she was diagnosed as having immune-mediated thrombocytopenia caused by the PEG-IFN-α-2a therapy. Therefore, the PEG-IFN/RBV was discontinued, but the platelet count remained below 1.0 × 10^4^/μl. We confirmed that her anti-Helicobacter pylori was positive but did not perform eradication therapy. In March 2012, 60 mg of prednisolone were administered orally. After starting corticosteroid therapy, the platelet count increased gradually. Then the prednisolone was gradually tapered down by 10 mg every 4 days, the platelet count gradually rose above 10 × 10^4^/μl, and the PAIgG was decreased to 80 ng/L. When the prednisolone dosage decreased to 30 mg per day orally, she was discharged. The prednisolone was gradually tapered off, and the platelet count returned to normal (Figure 2). Prednisolone was discontinued in May 2012. Her platelet count remained normal during the follow-up. And HCV RNA has never been detected since. She was diagnosed as having a sustained virologic response (SVR) 6 months later.

## Discussion

To our knowledge, this is the first case report to assess the relationship between severe thrombocytopenia that developed in a patient treated with PEG-IFN/RBV and that patient having the *ITPA* genotype. It is a worthwhile case because, in spite of having the *ITPA-CC* genotype, the patient developed severe thrombocytopenia caused by the PEG-IFN/RBV treatment.

It is generally thought that an *ITPA* genetic variant is independently associated with reduction in the mean platelet counts for weeks 2, 4, 8, and 12 [5]; while it is also associated with the reduction of hemoglobin for those same weeks caused by the RBV [5]. On the other hand, *IL28B* genetic variant strongly associated with the response to PEG-IFN/RBV [6]. Patients with the *IL28-TT* genotype exhibit higher prevalence of virologic response compared to those with *IL28B-TC/CC*[6]. When we began to administer the PEG-IFN/RBV treatment, we expected the possibility of SVR and had been cautious of the reduction of hemoglobin because the patient had the *IL28B-TT* and *ITPA-CC* genotypes. Indeed, she achieved negative HCV RNA after only 9 weeks administration of PEG-IFN/RBV and her hemoglobin level had gradually decreased to a maximum of 9.6 g/dl. The side effects of PEG-IFN/RBV treatment are widely known, such as leukopenia, anemia, and thrombocytopenia. Hematologic abnormalities often lead to dose reduction and premature withdrawal from therapy in 10%–14% of patients [7]. It is not rare that mild-to-moderate thrombocytopenia is caused by PEG-IFN/RBV, and it has been attributed to a direct inhibition of stem cell proliferation and differentiation in the bone marrow [8]. Yamane *et al*. reported IFN-α directly inhibited cytoplasmic maturation and platelet production but not proliferation or endomitosis in human primary megakaryocytes [9]. However, severe thrombocytopenia (platelet counts less than 2.5 × 10^4^/mm^3^) during interferon therapy is rare, and it is a life-threatening side effect.

In the present case, in the early period of administration (within 12 weeks) of PEG-IFN/RBV, mild-to-moderate thrombocytopenia was caused by the treatment, but the platelet count remained at or around 10 × 10^4^/μl for 9 months. Li *et al*. reported in their review of severe thrombocytopenia induced by IFN-α in 17 patients [10] that the median onset time from the administration of IFN-α/PEG-IFN-α treatment was 3.6 months (range, 1–36 months), and the patients could recover by discontinuing the IFN-α/PEG-IFN-α and administering an immunosuppressant. It is noteworthy that they reported that 16 patients (94%) had reached SVR in spite of a short period of IFN-α administration. Generally, PEG-IFN/RBV can achieve rates of an SVR of less than 50% in patients with *HCV* genotype *1*[2]. Therefore, there might be a correlation between severe thrombocytopenia and SVR that is caused by PEG-IFN/RBV therapy.

It is necessary to consider the mechanism in the present case of this severe thrombocytopenia. The IFN-α treatment is considered an immune-modulator and increases the risk for immune thrombocytopenia purpura in patients with hepatitis C [10], but the mechanism of IFN-induced autoimmune thrombocytopenia is unclear [10]. IFN-α/PEG-IFN-α can induce the production of autoantibody against thrombocytes, such as platelet antibody, which can lead to immune-mediated thrombocytopenia [11]. This situation is critical and may sometimes result in death. IFN can enhance the expression of major histocompatibility class I antigens and promote the production of IL-1 (interleukin 1) and TNF-α (tumor necrosis factor alpha). The over expression of these cytokines can induce autoimmune disease [12]. It is observed that higher Th1 (T helper 1) cell reactivity with platelets is related to idiopathic thrombocytopenic purpura (ITP) patients. PEG-IFN can increases IFN-γ secretion and improve CD4 T cell response in HCV-infected patients [13]. This may be one of the reasons of autoimmune thrombocytopenia during PEG-IFN treatment [14]. Recently, it is considered that autoimmune thrombocytopenia is associated with imbalance of Treg/Th17 cells [15]. But, to our knowledge, there are no data in PEG-IFN induced thrombocytopenia.

Regarding the treatment, we discontinued PEG-IFN/RBV after we diagnosed IFN-induced autoimmune thrombocytopenia and administrated 60 mg of prednisolone first. Li *et al*. reported that early administration of immunosuppressant was an effective therapy for IFN-α induced severe thrombocytopenia [10].

Finally, it was considered that this case was “possible” drug-induced immune thrombocytopenia based on criteria and level of evidence for establishing a causative relationship in drug-induced thrombocytopenic purpura by George *et al*. [16,17]. It is the reason that this case meets only their first criteria and evidence: therapy with the candidate drug preceded the thrombocytopenia, and recovery from thrombocytopenia was complete and sustained after discontinuation of therapy.

The 2011 practice guideline by the AASLD (American Association for the Study of Liver Diseases) recommended that *IL28B* genotype variants are robust pretreatment predicators of the SVR to PEG-IFN/RBV in patients with genotype *1* chronic HCV infection [1]. In thrombocytopenia caused by PEG-IFN/RBV, there is a tendency that patients with the *ITPA-CC* genotype are considered to be less at risk than those with the *ITPA-CA/AA* genotype. However, the newest treatment (PEG-IFN/RBV and protease inhibitors) might weaken this tendency. Therefore, this case of severe thrombocytopenia that developed in a patient with chronic hepatitis C treated with PEG-IFN/RBV in spite of the patient having the *ITPA-CC* genotype should be kept in mind.

## Conclusion

In conclusion, it is generally understood that the *CC* genotype had significantly less reduction in the mean platelet counts compared with the *AA/CA* genotype. However, we should pay careful attention in the differential diagnosis for patients with the *ITPA-CC* genotype because, although rare, severe thrombocytopenia could occur.

## Consent

Written informed consent was obtained from the patient for publication of this Case Report and any accompanying images. A copy of the written consent is available for review by the Editor-in-Chief of this journal.

## Competing interest

The authors declare that they have no competing interests.

## Authors’ contributions

WJ and HH gathered the information for this case and were the major contributors in writing the manuscript. TN contributed to the writing in the discussion section and the genome sequence techniques. HK and WK contributed to the writing in the discussion section. All authors read and approved the final version of the manuscript.
